# Epigenetic modifications in bladder cancer: crosstalk between DNA methylation and miRNAs

**DOI:** 10.3389/fimmu.2025.1518144

**Published:** 2025-02-05

**Authors:** Wei Li, Peiyue Luo, Qi Chen, Le Cheng, Lifeng Gan, Fangtao Zhang, Haidong Zhong, Liying Zheng, Biao Qian

**Affiliations:** ^1^ The First Clinical College, Gannan Medical University, Ganzhou, Jiangxi, China; ^2^ Department of Urology, The First Affiliated Hospital of Gannan Medical University, Ganzhou, Jiangxi, China; ^3^ Key Laboratory of Urology and Andrology of Ganzhou, Ganzhou, Jiangxi, China; ^4^ Department of Graduate, The First Affiliated Hospital of Gannan Medical University, Ganzhou, Jiangxi, China

**Keywords:** bladder cancer, epigenetic modification, DNA methylation, microRNA, cancer therapy

## Abstract

Bladder cancer (BC) is a malignant tumor characterized by a high incidence of urinary system diseases. The complex pathogenesis of BC has long been a focal point in medical research. With the robust development of epigenetics, the crucial role of epigenetic modifications in the occurrence and progression of BC has been elucidated. These modifications not only affect gene expression but also impact critical biological behaviors of tumor cells, including proliferation, differentiation, apoptosis, invasion, and metastasis. Notably, DNA methylation, an important epigenetic regulatory mechanism, often manifests as global hypomethylation or hypermethylation of specific gene promoter regions in BC. Alterations in this methylation pattern can lead to increased genomic instability, which profoundly influences the expression of proto-oncogenes and tumor suppressor genes. MiRNAs, as noncoding small RNAs, participate in various biological processes of BC by regulating target genes. Consequently, this work aims to explore the interaction mechanisms between DNA methylation and miRNAs in the occurrence and development of BC. Research has demonstrated that DNA methylation not only directly influences the expression of miRNA genes but also indirectly affects the maturation and functionality of miRNAs by modulating the methylation status of miRNA promoter regions. Simultaneously, miRNAs can regulate DNA methylation levels by targeting key enzymes such as DNA methyltransferases (DNMTs), thereby establishing a complex feedback regulatory network. A deeper understanding of the crosstalk mechanisms between DNA methylation and miRNAs in BC will contribute to elucidating the complexity and dynamics of epigenetic modifications in this disease, and may provide new molecular targets and strategies for the early diagnosis, treatment, and prognostic evaluation of BC.

## 1 Introduction

Bladder cancer (BC), one of the most lethal malignancies of the urinary tract, ranks as the ninth most commonly diagnosed cancer worldwide. According to GLOBOCAN data statistics, approximately 614,000 new cases and 220,000 deaths were reported in 2022 (1). While the geographical distribution of BC incidence remains a subject of debate, it is projected that both mortality and morbidity will continue to rise in the coming decades (2, 3). Consequently, the costs associated with monitoring and preventive interventions for BC are likely to increase persistently, significantly impacting the quality of life for patients and imposing a financial burden (4). BC encompasses a diverse array of pathological types, including urothelial carcinoma, squamous cell carcinoma, and adenocarcinoma, with urothelial carcinoma being the most prevalent (5). BC can be classified into invasive and non-invasive categories based on the depth of tumor invasion. Among these, non-muscle invasive BC (NIMBC) presents a low risk and has a favorable prognosis, whereas muscle invasive BC (MIBC) is associated with an unfavorable prognosis. Approximately 75% of BC patients are diagnosed with NIMBC, which has a relatively favorable prognosis, with a 5-year survival rate of as high as 43% (6). Adjuvant chemotherapy is recommended to improve the survival rate of MIBC patients, although the 5-year survival rate for this group is only 4.8% (7). Since the 1990s, the treatment of BC has remained relatively stable overall. Clinically, transurethral resection, chemotherapy, radiotherapy, and immunotherapy are predominantly employed for comprehensive management (8). Currently, the prognosis for patients with BC is closely linked to the regulation of the immune system. Certain immune and inflammatory biomarkers, such as the systemic immune inflammation index, can effectively predict patient outcomes (9). However, approximately 60%-70% of BC patients experience relapse following treatment, and some individuals demonstrate resistance to radiotherapy and chemotherapy (10). Therefore, an in-depth exploration of the molecular mechanisms underlying the occurrence and progression of bladder tumors is crucial for enhancing prevention and optimizing clinical diagnosis and treatment strategies.

Epigenetics, a term initially proposed by the British developmental biologist Conrad Waddington, encompasses all molecular pathways through which the expression of genotype is regulated to produce a specific phenotype (11). With advancements in research, the focus of epigenetics has shifted primarily to stable and heritable variations in gene expression during mitosis and meiosis, occurring without alterations in DNA sequences (12). Key epigenetic modifications include DNA methylation, histone post-translational modifications, chromatin remodeling, and regulation induced by noncoding RNAs. Aberrant epigenetic modifications have been closely linked to various diseases, including autoimmune disorders, metabolic conditions, and cancer (13–15). Numerous studies have demonstrated that these aberrant modifications can influence the onset and progression of cancer by modulating factors such as proliferation, migration, angiogenesis, and the immune microenvironment of cancer cells (16). Furthermore, research has shown a significant relationship between aberrant DNA methylation and miRNA expression in BC. For example, the upregulation of ITGA8 methylation in cells can significantly promote the proliferation and metastasis of BC cells. Conversely, the inhibition of ITGA8 methylation can enhance the expression of ITGA8 mRNA, allowing a greater number of cancer cells to remain in the G0/G1 phase (17). Furthermore, miR-299-5p can facilitate the development of BC by targeting and inhibiting DOX7 expression (18). A comprehensive study has revealed a complex relationship between DNA methylation and microRNAs (miRNAs) in the progression of BC. High levels of methylation in gene promoters can silence the expression of associated miRNAs, thereby impacting BC. Specifically, elevated methylation of the miR-1-3p CpG island leads to a decrease in miR-1-3p expression in BC, which accelerates the depletion of glutaminase and promotes the proliferation, migration, and invasion of BC cells (19). On the other hand, miRNAs can modulate DNA methylation by targeting DNMT enzymes and can also regulate DNA methylation by modulating methylation-related regulators, thus affecting the progression of BC (20, 21). Additionally, the methylation of long noncoding RNA (lncRNA) promoters can target specific miRNAs through a “sponge effect,” thereby regulating miRNA expression and influencing BC progression (22). Thus, DNA methylation and miRNAs play critical roles in the advancement of BC, and intricate regulatory relationships exist between them.

In light of the numerous studies on DNA methylation or miRNA and BC progression, the mechanism of interaction between DNA methylation and miRNA remains ambiguous. The response of bladder tumors to drug therapy and radiotherapy is likewise regulated by the DNA methylation-miRNA network. Consequently, this review endeavors to examine the crucial role of DNA methylation and miRNAs in the progression of BC and to explore the reciprocal regulation of DNA methylation and miRNAs to provide novel ideas and strategies for the prevention, diagnosis, and treatment of BC.

## 2 Overview of DNA methylation and microRNAs

### 2.1 Introduction to the biological functions of microRNAs

**Figure 1:**
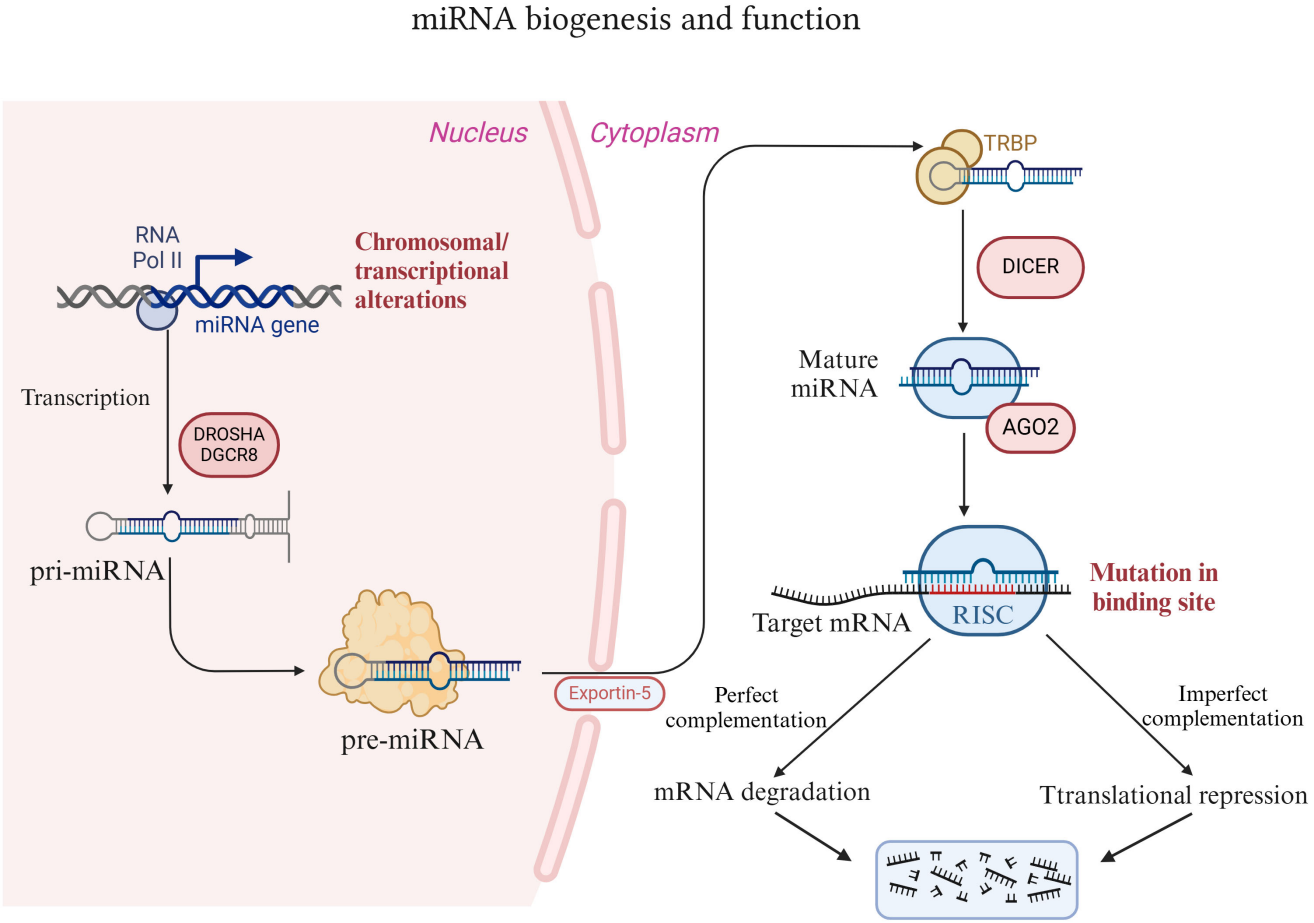
Introduction of miRNA biogenesis and function. MiRNAs are synthesized and processed within the nucleus. These proteins are subsequently exported to the cytoplasm via the XPO5 protein and further processed and matured by the nuclease DICER1. Thereafter, miRNAs bind to AGO2 to form a functional miRNA-induced silencing complex (miRISC), which in turn binds to the target mRNA to exert its effects.

There are only approximately 20,000 protein-coding genes in the human genome, accounting for less than 2%. Scientists have postulated that a large portion of the remaining human genome is nonfunctional and was initially regarded as “junk DNA” (23). Nevertheless, studies relying on advanced technologies such as RNA deep sequencing have revealed that many RNA transcripts exist (24). These transcripts do not originate from known genomes and do not encode proteins; hence, these RNAs are termed noncoding RNAs (ncRNAs). There are numerous kinds of ncRNAs, among which microRNAs (miRNAs) are the most extensively studied (25). MiRNAs constitute a class of endogenous noncoding RNA molecules that are prevalent in eukaryotes and are approximately 18-22 nucleotides in length (26). MiRNA biogenesis is an extremely conserved and meticulously regulated process (**Figure 1**). In mammals, the production of miRNAs commences with the transcription of RNA polymerase II (pol II) in the nucleus, yielding longer primary transcripts (pri-miRNAs) (27). These pri-miRNAs typically possess a cap structure (7MGpppG) and a polyadenylation tail (AAAAA), with a length of several thousand nucleotides (27). pri-miRNAs are subsequently recognized and cleaved by a microprocessor (comprising DROSHA and DGCR8) to generate pre-miRNAs with a stem−loop structure. The length of pre-miRNA is approximately 60-70 nucleotides, and it is the precursor form of miRNA (28). Pre-miRNAs are exported to the cytoplasm in a RAN-GTP-dependent manner via the transporter XPO5 and are further cleaved by the nuclease DICER1 to form double-stranded mature miRNAs (29, 30). In the double-stranded miRNA, the chain with low thermodynamic stability at the 5’ end is retained as the guide chain, whereas the other complementary chain is degraded.

The function of miRNAs lies primarily in recognizing target mRNAs through complementary base pairing and guiding the silencing complex to degrade target mRNAs or inhibit the translation of target mRNAs, depending on the degree of complementarity. Typically, miRNA binds to the 3’ untranslated region (3’ UTR) of the target mRNA and either degrades or inhibits the translation of the target mRNA. However, in rare instances, it can facilitate the translation of the target gene and take part in posttranscriptional gene regulation (31, 32). Specifically, mature miRNAs in the cytoplasm bind to proteins such as AGO2 to form functional miRNA-induced silencing complexes (miRISCs) (33). The nucleotide sequence responsible for the core function of miRNAs is termed the “seed sequence”, which recognizes and binds to the 3’ UTR of the target mRNA under the guidance of AGO2 (34). When the “seed sequence” is fully complementary to the target mRNA, the target mRNA is degraded. Conversely, when the “seed sequence” is not fully complementary, the translation of the target mRNA is inhibited (35, 36). This regulatory mechanism of miRNAs forms a complex “many-to-many” regulatory network in organisms. That is, one miRNA can regulate multiple mRNAs, and one mRNA can likewise be targeted by multiple miRNAs. Hence, the diversity of miRNA functions is being explored, and miRNAs play a significant role in the development of diseases. Research has revealed that miRNA expression is correlated with various cancers, and approximately 50% of annotated miRNAs are located in tumor-related fragile sites in the genome. These miRNAs play a vital role in tumorigenesis and have functions analogous to those of tumor suppressor genes and oncogenes (37–39). For example, miR-15a/miR-16-1 negatively regulate the antiapoptotic gene BCL2. Deletion or downregulation of miR-15a/miR-16-1 promotes the occurrence of leukemia and lymphoma (40, 41).

### 2.2 Overview of DNA methylation

**Figure 2:**
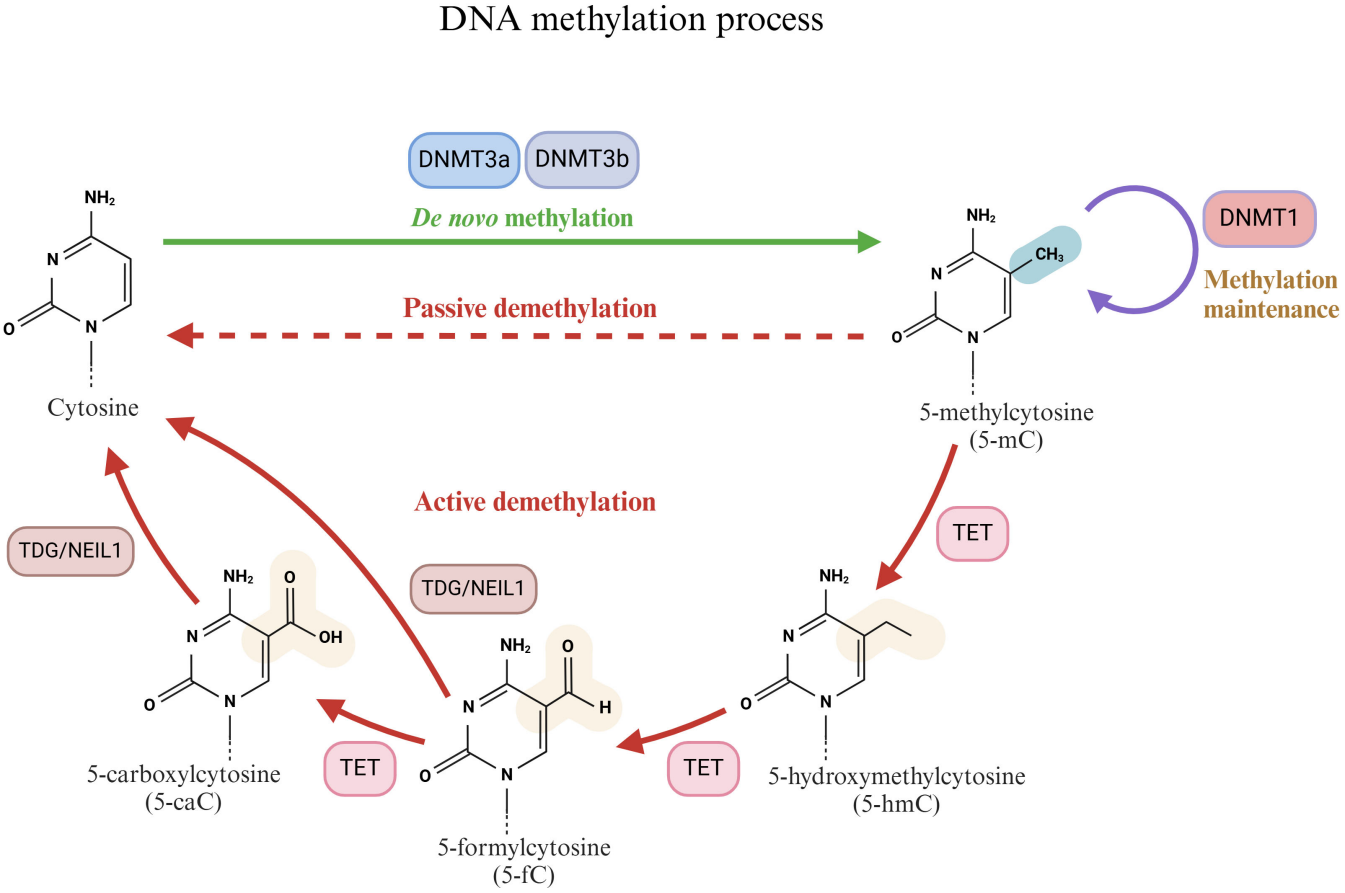
Schematic diagram of the DNA methylation process. Cytosine is methylated by DNMTs (DNMT1, DNMT3a and DNMT3b) to produce 5-methylcytosine (5-mC). 5-mC can be reduced to cytosine by active demethylation and passive demethylation. The active demethylation process is closely related to 10-11 translocases (TETs).

Mammalian DNA epigenetic modifications, specifically DNA methylation, are crucial regulators of organism development and key regulators of disease mechanisms. DNA methylation involves a methyl modification on the fifth carbon of cytosine (5-methylcytosine, 5mC) without altering the DNA sequence. It is typically present in the context of mammalian symmetrical CpG dinucleotides (42, 43). Approximately 70–80% of the CpG sites in the mammalian genome are methylated, yet they do not encompass specific regions known as CpG islands (CGIs) (44). CGI is a CpG-rich sequence with a length of approximately 1 kb in the gene promoter (45). Methylation within the gene body typically represents the upregulation of gene expression. However, aberrant methylation in CGIs, potentially due to DNA damage, usually signifies transcriptional repression (43, 46, 47). In the process of DNA methylation, the DNMT writer (DNA methyltransferase), MBD reader (methylated CpG binding domain protein), and TET modifier (TET enzyme family) play crucial roles (**Figure 2**).

#### 2.2.1 DNMT writers

DNA methyltransferases (DNMTs) are crucial enzymes that catalyze DNA methylation reactions (48). They primarily transfer the methyl group from S-adenosylmethionine (SAM) to the cytosine residue of DNA to generate 5mC. On the basis of different functions, DNMTs can be classified into several types, among which DNMT1, DNMT3a, and DNMT3b are the most significant. The primary function of DNMT1 is to preserve the existing DNA methylation pattern. During DNA replication, DNMT1 can recognize CpG sites on semimethylated DNA double strands and add methyl groups to newly synthesized strands to ensure the faithful transmission of DNA methylation patterns (49, 50). Conversely, DNMT3a and DNMT3b are classified as *de novo* methyltransferases and are involved in the *de novo* methylation process of DNA. They exhibit a strong affinity for unmethylated DNA and extensively methylate DNA in the early stages of embryonic development and cell differentiation (51, 52). DNMT3a is associated with the establishment of parental imprinting, whereas DNMT3b is related to chromosome structure (51, 53).

#### 2.2.2 MBD reader

DNA methylation is linked to other mechanisms, such as transcriptional repression at an early stage, which is accomplished by the binding of members of the MBD (methylated CpG binding domain) protein family. The MBD protein family can decipher the genome (hydroxy) methylation group and play a mediating role among cytosine modification, DNA and histone modification enzyme complexes, noncoding RNA, and different chromatin states. The protein family comprises 11 members, namely, MeCP2, MBD1 to MBD6, SETDB1, SETDB2, BAZ2A, and BAZ2B (54–56). In addition to the methyl-CpG binding domain (MBD), which shares the core functional region, all members of the family possess a variety of other highly specific domains that collectively endow them with unique DNA- and protein-binding properties and diverse functions. MeCP2 is the first protein in the MBD protein family that selectively binds to symmetrically methylated CpG dinucleotides (57). As a crucial regulator of nervous system development and function, MeCP2 not only recognizes methylated DNA via its N-terminal MBD but also interacts with transcriptional coinhibitory complexes (such as Sin3A/HDAC) through its C-terminal domain to repress gene expression (57, 58).

#### 2.2.3 TET modifier

DNA methylation typically inhibits gene transcription, whereas DNA demethylation can reactivate gene transcription via active or passive processes (59, 60). Passive DNA demethylation takes place during DNA replication. If the maintenance of the methylation of semimethylated DNA is impeded, such as a decrease in DNMT enzyme activity or a lack of SAM (methyl donor), methylation markers gradually vanish over time (61). In contrast to passive DNA demethylation, active DNA demethylation is an enzymatic process that is independent of DNA replication (62). Active DNA demethylation is intimately related to the 10-11 translocation (TET) enzyme family. The TET enzyme family, comprising three major members, namely, TET1, TET2, and TET3, is a distinctive class of dioxygenases that specifically recognize and bind to methylated cytosine (5-mC) sites on DNA molecules (62, 63). The TET enzyme subsequently employs its catalytic activity to initiate the oxidation reaction, transforming 5-mC into more active intermediates within CpG dinucleotides, such as 5-hydroxymethylcytosine (5-hmC), 5-formylcytosine (5-fC), and 5-carboxycytosine (5-caC) (64, 65). The generation of these intermediates not only signifies the transformation of the DNA methylation status but also offers an entry point for subsequent DNA repair mechanisms. Following the oxidation reaction, these oxidation products on the DNA molecule become the targets of DNA repair enzymes such as thymine DNA glycosylase (TDG) or Nei-like 1 (NEIL1) glycosylase (66, 67). They eliminate the modified bases via the base excision repair (BER) pathway and replace them with unmodified cytosine, thus achieving DNA demethylation (68).

## 3 Abnormal DNA methylation in BC

Although DNA methylation does not alter the fundamental sequence of DNA, it can modulate gene expression by influencing gene transcriptional activity. As a common genetic modification throughout the genome, DNA methylation occurs in promoters, enhancers, gene bodies, and intergenic regions (69). Consequently, DNA methylation plays a crucial role in biological processes such as cell differentiation, development, and disease occurrence. The process of tumor development involves complex methylation characteristics in comparison with those of nontumor tissues. In general, the cancer epigenome is characterized by overall hypomethylation. From normal cells to cancer cells, the methylation level decreases from 80% to 40–60%, whereas the DNA of CpG islands is hypermethylated (70, 71). This has given rise to a series of early cancer events, such as the overexpression of proto-oncogenes, increased mutation rates, chromosomal instability, and the silencing of tumor suppressor genes (72, 73). Specifically, this process facilitates invasion, metastasis, and angiogenesis during the development of various cancers, including BC.

### 3.1 DNA methylation and BC proliferation, invasion and migration

Conventional bladder epithelial cells exhibit a normal proliferation cycle, with their proliferation rate stringently regulated to ensure the appropriate renewal and repair of the bladder mucosa. In contrast, BC cells possess unlimited proliferative capacity, enabling rapid growth, increased spatial occupation, and enhanced invasion and migration capabilities. These cells can invade surrounding tissues and metastasize to distant organs via the bloodstream or lymphatic system. Studies have demonstrated that abnormal DNA methylation is closely associated with the malignant proliferation, invasion, and metastasis of BC cells during disease progression (74–76). Abnormal DNA methylation compromises genomic integrity, making the genome more susceptible to recombination and mutation, while also activating a series of genes involved in cell proliferation, apoptosis, invasion, and metastasis. Ma et al. utilized CHARM analysis to investigate the methylation profile in BC tissues, revealing a significant elevation in the methylation level of ITGA8, which was negatively correlated with its expression level, thus promoting the proliferation and migration of BC cells (17). Additionally, hypomethylation of the CLDN4 promoter is correlated with the malignant phenotype of BC, contributing to increased cell proliferation, stemness, and epithelial-mesenchymal transition (EMT) (74). Furthermore, certain tumor suppressors in BC are closely linked to DNA methylation. For instance, CYGB exhibits antitumor effects across various human malignancies; however, its expression is often diminished in tissues associated with ovarian cancer, lung cancer, and breast cancer (77–79). In BC, the sex-determining region Y-box 7 (SOX7) binds to the promoter of DNMT3b, leading to its transcriptional inhibition. This results in decreased methylation of the CYGB promoter and increased expression of CYGB, ultimately inhibiting the proliferation, migration, and invasion of BC cells (76). Further studies have revealed that alterations in the tumor immune microenvironment induced by DNA methylation are closely associated with the progression of BC. Macrophages (MACs) are typically linked to poor prognosis within the tumor microenvironment (TME) due to their frequent reprogramming into immunosuppressive cells (80). In a recent study, Fabregat et al. reported that the immune functionality of macrophages can be reprogrammed under hypoxic conditions. Activated macrophages (mMAC1) display enhanced proinflammatory properties, characterized by elevated levels of IL-6 and TNF-α, alongside reduced levels of IL-10 (81). Mechanistically, the immunogenicity of mMAC1 is driven by DNA demethylation mediated by the specific proinflammatory gene NF-κB, rather than TET2. In patients with BC, tumors characterized by a higher load of mMAC1 cells are associated with improved overall survival, suggesting that mMAC1 may counteract tumors by bolstering the immune response (81). In summary, this study demonstrates that macrophages can be reprogrammed into proinflammatory cells through DNA demethylation under hypoxic conditions. These cells not only refrain from suppressing immunity within the BC microenvironment but may also enhance antitumor immunity by promoting the T-cell response, thereby offering a new perspective and potential target for BC treatment.

### 3.2 DNA methylation and treatment of BC

For the majority of MIBC patients, adjuvant chemotherapy is currently one of the most widely recognized treatments for prolonging survival (7). However, the resistance of BC cells to chemotherapeutic drugs presents a prevalent challenge. Therefore, understanding the mechanisms underlying drug resistance in BC is essential. DNA methylation plays a distinctive role in influencing the drug resistance of BC cells. An analysis of the TCGA BC database revealed that RRBP1 is an oncogene associated with poor prognosis and is highly expressed in advanced and lymphatically metastatic tumor tissues (75). MS-PCR was employed to assess the methylation levels of RRBP1 in both normal cell lines and cancer cell lines (RT4, T24, J82, and BFTC909). Overall, the methylation levels in cancer cell lines were found to be decreased. Notably, the methylation levels of advanced BC cell lines (T24 and J82) were lower. Consequently, the poor prognosis observed in advanced patients may be attributed to low methylation and significantly increased expression of the RRBP1 promoter (75). Luo et al. collected and cultivated organoids from tumor patients and conducted drug sensitivity tests. They discovered that tumor organoids treated with cisplatin, gemcitabine, and epirubicin exhibited reduced reactivity to chemotherapy when RRBP1 expression was higher (75). These findings suggest that regulating the methylation level of RRBP1 may represent a potential target for enhancing BC resistance. D-3-Phosphoglycerate dehydrogenase (PHGDH) plays a crucial role in serine synthesis. However, the PHGDH gene is frequently amplified in various cancers, including BC (82, 83). Notably, the expression level of PHGDH in high-grade BC patients was significantly higher than that in low-grade BC patients, and the survival rate of patients with elevated PHGDH expression decreased markedly. This phenomenon may be associated with the DNA copy number of PHGDH and its hypomethylation (83). Consequently, regulating PHGDH expression could represent a potential therapeutic strategy for BC. A study conducted by Yoshino et al. supported these findings, demonstrating that the downregulation of PHGDH expression through siRNAs or inhibitors significantly inhibited proliferation and induced apoptosis in BC cell lines. Furthermore, the combination of PHGDH inhibitors with gemcitabine or cisplatin resulted in synergistic tumor inhibition both *in vitro* and *in vivo* (83). Additionally, p73, a homolog of p53, serves as a determinant of chemosensitivity (84). Bunch et al. utilized the Illumina 450K methylation chip to analyze over 150 BC patient samples, identifying 12 distinct CpG sites within the p73 gene locus. Compared to adjacent normal tissues, these sites were found to be hypermethylated in tumors (85). Patients exhibiting high p73 promoter methylation at the P1 CpG site (cg07382920) demonstrated a lower survival rate. *In vitro* studies utilizing the DNA demethylation agent decitabine (DAC) revealed that it reduced TAp73 methylation and upregulated its expression, significantly enhancing the sensitivity of T24 and CR-T24 (cisplatin-resistant T24 cells) to cisplatin (85). In addition to chemotherapy, immunotherapy is associated with DNA methylation. Bacillus Calmette-Guerin (BCG) immunotherapy serves as the standard adjuvant treatment for certain moderate-and high-risk NMIBC patients. However, the response rate varies significantly and is influenced by numerous factors (86). Ilijazi et al. conducted a genome-wide DNA methylation analysis to compare the differences between the BCG response group and the BCG failure group. They identified six differentially methylated CpG sites located in the promoters of the genes *GPR158, KLF8, C12orf42, WDR44, FLT1, and CHST11* (87). Functional verification through bisulfite sequencing revealed that hypermethylation of the GPR158 promoter was the most reliable predictor of BCG failure (87). Similarly, the fibrinogen-related protein family member FGL2 is downregulated in BC due to hypermethylation in its promoter region, which has been correlated with poor prognosis. Notably, BC patients with high expression of FGL2 demonstrated a better response to immunotherapy (88).

In conclusion, some tumor-related genes in BC are unbalanced due to abnormal DNA methylation, thereby facilitating malignant behaviors such as tumor proliferation, migration, chemotherapy resistance, and immune tolerance (**Table 1**). Targeted regulation of DNA methylation to influence the progression of BC or enhance drug tolerance during treatment may be an important target for future research.

**Table 1:** Effects of DNA methylations on BC.

Gene	State of methylation	The impact on BC	Ref
ITGA8	Hypermethylation	Promote the proliferation, migration and invasion of BC cells.	(17)
CLDN4	Promoter hypomethylation	Induce decreased apoptosis of BC cells, increased drug resistance and increased cancer metastasis potential.	(74)
RRBP1	Hypomethylation	Promote the growth of BC cells and chemotherapy resistance.	(75)
CYGB	Promoter hypomethylation	Inhibit the proliferation, migration and invasion abilities of BC.	(76)
NF-κB	Hypomethylation	Promote macrophages to secrete pro-inflammatory factors (IL-6 and TNF-α), thereby enhancing the immune response.	(81)
PHGDH	Hypomethylation	Inhibit the proliferation ability of BC cells and induce apoptosis.	(83)
TAp73	Hypermethylation	Increase the cisplatin response of BC cells.	(85)
GPR158	Promoter hypermethylation	GPR158 is the best predictor of BCG failure.	(87)
FGL2	Promoter hypermethylation	BC patients with high expression of FGL2 show a better response to immunotherapy.	(88)
GSTM2	Hypermethylation	Promote apoptosis, anti-proliferation, anti-inflammation and anti-angiogenesis of BC cells.	(89)
GSTM5	Promoter hypermethylation	Accelerate GSH depletion, thereby inhibiting the proliferation and migration of BC cells.	(90)
GULP1	Promoter hypermethylation	Disrupt the KEAP1-NRF2 pathway, thereby promoting BC cell proliferation and cisplatin resistance.	(91)
FOXA1	Hypermethylation	Drive squamous differentiation of BC and increase the sensitivity to specific carcinogens.	(92)
KLF11	Hypermethylation	Promote the adhesion of abnormal cancer cells to the basement membrane and promote the occurrence of papillary urothelial carcinoma.	(93)
SLC31A1	Hypomethylation	Promote the proliferation of BC cells by regulating processes related to copper metabolism homeostasis.	(94)
LOXL1	Hypermethylation	Inhibit the level of E-cadherin and initiate the EMT process.	(95)

## 4 Role of abnormally expressed miRNAs in BC

Since noncoding RNA molecules are continuously produced in cells, their role has been investigated. Although initially regarded as a “junk factor”, further investigations have shown that noncoding RNA molecules (especially miRNAs) play crucial roles, ranging from affecting cell growth and migration to regulating apoptosis, autophagy, differentiation, and important cell death mechanisms (96–98). During the progression of BC, the expression of miRNAs is frequently dysregulated. In-depth investigations have revealed that miRNAs play a significant role in the proliferation, invasion, migration, and drug resistance of BC. Consequently, in this section, we concentrate on the relevant biological mechanisms.

### 4.1 MiRNAs and BC proliferation and metastasis

The specific role of miRNA in the development of BC remains unclear, exhibiting a dual role as both cancer-promoting factors and tumor suppressors. This duality primarily depends on their targeted genes, the involved signaling pathways, and the tumor microenvironment. Current studies indicate that the expression of most miRNAs tends to decrease in various cancers. Increasing the expression of these miRNAs can inhibit the proliferation, survival, and migration of cancer cells, thereby acting as tumor suppressors. Notably, low expression levels of miR-3619-5p in BC samples facilitate the growth and invasion of cancer cells (99). Conversely, exogenous overexpression of miR-3619-5p in BC cells effectively inhibits tumor cell proliferation, migration, and invasion. Mechanistically, miR-3619-5p suppresses cancer cell growth and metastasis by targeting its promoter to activate p21, while concurrently reducing the expression levels of β-catenin and CDK2 (99). Zhao et al. reported that low levels of miR-133b and its target gene transgelatin 2 (TAGLN2) are closely associated with decreased survival rates in patients with BC (100). TAGLN2 is aberrantly expressed in various cancers, including gastric and esophageal cancers, with high expression correlating with increased malignancy and metastatic potential (101, 102). Further investigations have revealed that upregulation of miR-133b expression in BC can inhibit a range of cancer-promoting processes, such as glucose uptake, invasion, angiogenesis, and colony formation, by targeting TAGLN2 (100). Similarly, the levels of miR-206, miR-1, miR-133a, and miR-145 were significantly decreased in bladder tumors. The overexpression of these miRNAs is advantageous for the development of BC (103–105). These findings suggest that certain miRNAs function as tumor suppressors in the progression of BC. Conversely, other miRNAs may act as tumor-promoting agents in BC by targeting and silencing associated tumor suppressor genes. DAB2IP is a well-known tumor suppressor (106). Huang et al. demonstrated that miR-92b can directly inhibit the expression of DAB2IP, thereby promoting the EMT of BC cells and enhancing their migration and invasion capabilities (107). Estrogen receptor β (ERβ) can upregulate the expression of miR-92a at the transcriptional level. miR-92a can diminish the expression of the DAB2IP tumor suppressor gene by binding to the miR-92a binding site located on the 3’UTR of DAB2IP, thus facilitating the growth and invasion of BC (108). Similarly, miR-20a-5p, which targets and silences NR4A3, miR-93, which targets and silences PEDF, and miR-20a, which targets and silences LASS2, can also promote the progression of BC (109–111). In conclusion, miRNAs are closely linked to the proliferation, invasion, and metastasis of BC. However, their specific mechanisms and effects require further investigation.

### 4.2 MiRNAs and treatment of BC

In the process of exploring the innovation and optimization of BC treatment strategies, the regulatory effect of miRNAs on BC drug resistance has emerged as a research hotspot. Given the phenomenon of drug resistance in BC treatment, miRNAs offer a new approach to overcoming BC drug resistance through their unique gene expression regulatory mechanism.

As early as 2009, Adam et al. reported that members of the miR-200 family (specifically, miR-200b and miR-200c) play crucial roles in regulating the EMT process and the sensitivity of BC cells to epidermal growth factor receptor (EGFR)-targeted therapy (112). In the mesenchymal phenotype of the UMUC3 cell line, stable expression of miR-200 can upregulate the level of E-cadherin while downregulating the expression of ZEB1, ZEB2, and ERRFI-1, thereby increasing sensitivity to EGFR blockers. In the UMUC5 and T24 cell lines, both silencing and overexpression of miR-200 or the target gene ERRFI-1 confirmed changes in the sensitivity of BC to EGFR treatment (112). This finding not only enhances our understanding of the mechanisms underlying drug resistance in BC but also presents a novel strategy for improving the therapeutic efficacy of EGFR blockers. Subsequent studies have focused on the role of microRNAs in BC resistance to specific chemotherapeutic agents, such as cisplatin. For instance, miRNA-27a regulates glutathione (GSH) biosynthesis by targeting SLC7A11, which leads to cisplatin resistance in BC cells (113). Similarly, the expression of miR-101 was found to be downregulated in the cisplatin-resistant T24 cell line. The use of COX-2 siRNA to inhibit COX-2 could reverse the cisplatin resistance induced by the downregulation of miR-101 (114). The absence of chemical resistance management in BC contributes to the emergence of multidrug resistance. Researchers have identified that miR-193a-3p plays a complex regulatory role in facilitating multidrug resistance in BC. Deng et al. initially reported that the expression of miR-193a-3p was elevated in chemotherapy-resistant BC cell lines (H-bc and UM-UC-3), with multiple rounds of chemotherapy resistance achieved by inhibiting the oxidative stress (OS) pathway via the lysyl oxidase-like 4 (LOXL4) gene (115). Through literature mining and analysis of protein-protein interaction databases, they proposed five LOXL4-integrating protein chaperones (SUV39H1, CDC37, ECSIT, EXOC6, and COL2A1) as potential key components of the OS pathway’s response to damage signals, such as chemotherapy (115). Furthermore, their team discovered that miR-193a-3p can promote multidrug resistance in BC through various genes, including HOXC9, PSEN1, and ING5 (116–118). These findings not only highlight the intricate role of miRNAs in the mechanisms underlying drug resistance in BC but also provide a theoretical foundation for the development of novel treatment strategies targeting drug resistance.

Currently, the response rate of BC patients to immunotherapy remains relatively low. Given the significant role of microRNAs (miRNAs) in tumor growth, their involvement in regulating immune escape and sensitivity to immunotherapy has garnered considerable attention. Programmed death ligand-1 (PD-L1) is crucial for immune regulation and tumor immune evasion. Tsai et al. reported that the combination of the autophagy inhibitors chloroquine (CQ) and bafilomycin A1 (Baf-A1) can enhance the expression of PD-L1 in BC cells through the ERK-JNK-c-Jun signaling pathway (119). Mechanistically, miR-34a inhibits PD-L1 via this same signaling pathway. Furthermore, the overexpression of miR-34a in BC cells can prevent the increase in PD-L1 expression induced by autophagy blockers (CQ and Baf-A1) (119). Consequently, the combined administration of autophagy inhibitors and PD-L1 immune checkpoint blockade may represent a promising treatment strategy for BC. Additionally, Hui et al. identified three immunotherapy-related long non-coding RNAs (lncRNAs) based on the Imvigor210 database and BC expression data from the TCGA cohort (120). Following the establishment of an immunotherapy-related prognostic model, significant differences in immune cell infiltration and the effects of immunotherapy were observed between the high-risk and low-risk groups. According to the competing endogenous RNA (ceRNA) network theory, lncRNAs can regulate messenger RNA (mRNA) expression by sponging miRNAs. They constructed a ceRNA network involving lncRNA SBF2-AS1, miRNA hsa-miR-582-5p, and mRNA HNRNPA2B1. Additionally, they identified the eight small-molecule drugs with the highest affinity for the target protein HNRNPA2B1 (120). In conclusion, this study suggests that SBF2-AS1 may increase the expression of HNRNPA2B1 by sponging miR-582-5p, thereby promoting the occurrence and progression of BC and facilitating immune escape.

Following in-depth discussion of the multiple roles of miRNAs in the treatment of BC, it is evident that miRNAs, as important gene expression regulators, play essential roles in many key treatment aspects, such as enhancing the chemotherapy sensitivity of BC, deciphering the immune escape mechanism, and optimizing the immunotherapy strategy.

## 5 DNA methylation and miRNA crosstalk in BC

Current research has demonstrated that abnormal DNA methylation or disrupted miRNA expression is intimately associated with the progression of BC. These two factors involve numerous similar molecular mechanisms involved in the progression of BC, such as influencing tumor cell proliferation, invasion, and drug resistance. Further investigations revealed that abnormal methylation of miRNA promoters is relatively prevalent in BC, resulting in an imbalance in miRNA expression. This, in turn, regulates multiple aspects of BC, including tumor cell proliferation, invasion, migration, and the tumor microenvironment, and has an impact on treatment resistance (**Table 2**). Moreover, miRNAs can regulate the expression of oncogenes and tumor suppressor genes by targeting methyltransferase DNMTs and can also indirectly affect the development of BC. Additionally, a complex feedback mechanism exists between miRNAs and DNMTs. Abnormal methylation of miRNA promoters can disrupt the level of DNMTs, and an imbalance in DNMTs has a reciprocal effect on miRNA expression, thus forming a dynamic regulatory network that jointly affects the development of BC. Therefore, an in-depth understanding of epigenetic modifications during the progression of BC and the specific roles of DNA methylation and miRNAs can provide new perspectives for the treatment of BC in the future.

**Table 2:** Effects of aberrant methylation of the miRNA promoter on BC.

MicroRNA	Promoter status	Function	Ref
miR-1-3p	Hypermethylation	Inhibit the proliferation, migration and invasion of BC cells.	(19)
miR-34a	Hypermethylation	Induce BC cell senescence and cell cycle arrest.	(122)
miR-34a	Hypermethylation	Inhibit the proliferation and tumorigenicity of cancer cells and increase the cisplatin response.	(123)
miR-203	Hypermethylation	Increased apoptosis, thereby inhibiting the proliferation of BC cells.	(124)
miR-608	Hypermethylation	Inhibit tumor development by inducing G1 phase arrest through AKT/FOXO3a signaling.	(125)
miR-200b	Hypermethylation	Promote cisplatin resistance in BC cells.	(126)
miR-193a-3p	Hypomethylation	Promote multichemotherapy resistance of BC H-bc cell line.	(127)
miR-126	Hypermethylation	Inhibit the proliferation, migration and invasion of BC cells.	(128)
miR-200, miR-205	Hypermethylation	Promote EMT and invasion of BC.	(129)
miR-517a	Hypermethylation	Reduce apoptosis, thereby promoting the proliferation of BC cells.	(130)
miRs-137/124-2 /124-3/9-3	Hypermethylation	Ectopic expression of miR-137 or miR-124 inhibits BC cell proliferation, and ectopic expression of miR-9 inhibits BC cell invasion.	(131)
miR-300	Hypermethylation	Inhibit BC cell migration through the SP1/MMP9 pathway.	(132)
miR-888	Hypomethylation	Promote BC cell invasion.	(133)
miR-23a, miR-27a	Hypomethylation	Activate the Wnt signaling pathway by targeting SFRP1, thereby promoting the proliferation, migration and invasion of BC cells.	(134)
miR-145	Hypomethylation	Significantly associated with muscle-invasive disease and high-grade tumor staging in BC patients.	(135)
miRs-9/149/210/212/328/ 503/1224/1227/1229	Hypermethylation	Correlated with tumor grade, stage and prognosis.	(136)

### 5.1 Abnormal methylation of the miRNA promoter in BC

Transcription factors encoded by the tumor suppressor gene p53 have been shown to regulate microRNA expression, particularly inducing miR-34a and miR-34b/c, which suggests that these microRNAs are target genes of p53 (121). Lodygin et al. reported that high CpG methylation of the miR-34a promoter led to the silencing of its expression in several types of cancer, including BC (2/6, 33.3%) (122). Mechanistically, following DNA damage, the silencing of miR-34a surpasses its transactivation by p53. They re-expressed miR-34a in prostate and pancreatic cancer cell lines and found that miR-34a can induce senescence and cell cycle arrest by targeting CDK6, indicating that miR-34a functions as a tumor suppressor gene in tumors (122). Although this does not directly clarify the specific role of miR-34a in BC, it provides a valuable reference for future research. A subsequent study by Li et al. elucidated the precise biological role of miR-34a in BC (123). Through *in vivo* and *in vitro* experiments, the overexpression of miR-34a significantly inhibited the proliferation of MIBC cells, reduced their colony formation potential, and enhanced their sensitivity to cisplatin. At the mechanistic level, CD44 was identified as a target of miR-34a in MIBC cells. MiR-34a inhibits the transcription of CD44, thereby suppressing both the proliferation and chemosensitivity of MIBC cells. Furthermore, the overexpression of CD44 can effectively counteract the effects of miR-34a on tumor cell proliferation and chemosensitivity (123). In conclusion, the aforementioned studies demonstrate that abnormal methylation of the miR-34a promoter significantly impacts BC progression. Similarly, miR-203 is downregulated in BC due to promoter hypermethylation (124). Curcumin (diferuloylmethane), a natural compound recognized for its anticancer properties across various cancers, including BC, has been shown to influence miR-203 expression. Promoter methylation analysis revealed that curcumin treatment leads to partial demethylation of the miR-203 promoter in BC cell lines, resulting in increased expression of miR-203. Mechanistic studies indicate that curcumin induces hypomethylation and upregulation of the miR-203 promoter. Consequently, miR-203 targets and downregulates Akt2 and Src, thereby inhibiting BC cell proliferation, invasion, and migration while inducing apoptosis (124). Additionally, miR-608 is frequently downregulated in human BC tissues due to hypermethylation of its promoter. The upregulation of miR-608 expression in BC cells can induce G1 phase arrest via the AKT/FOXO3a signaling pathway, subsequently suppressing the proliferation and tumorigenesis of BC cells (125). In conclusion, the studies mentioned above demonstrate that abnormal methylation, particularly hypermethylation, of related miRNA promoters significantly impacts their expression in BC. This alteration can influence the development of BC by regulating tumor suppressors or associated signaling pathways related to proliferation, invasion, and migration.

Abnormal methylation of the miRNA promoter significantly influences the chemoresistance of BC. As previously mentioned, modulating the methylation status of the miR-34a promoter affects the sensitivity of BC cell lines to cisplatin. Similarly, Shindo et al. reported that the downregulation of CpG island hypermethylation of miR-200b enhances cisplatin resistance in T24 cells (126). Treatment of T24-resistant cells with 5-aza-dC markedly increased cisplatin toxicity, thereby suppressing tumor cell proliferation. Notably, most tumors, including BC, are currently susceptible to multidrug resistance. Lv et al. identified 5637 cells as the most sensitive cell line to various chemotherapies, while H-bc cells were found to be the most resistant cell line, as determined by measuring the drug dose that killed 50% of BC cells after 72 hours of treatment with pirarubicin (Pi), paclitaxel (Pa), adriamycin (Ad), and epirubicin hydrochloride (EH) (127). Biomic (miRomic and methylomic) analysis revealed that miR-193a-3p exhibited one of the highest degrees of differential expression and methylation among CpG islands. It was hypermethylated in 5637 cells, whereas there was minimal methylation observed in H-bc cells. MiR-193a-3p is capable of modulating multiple downstream targets that influence the chemoresistance of BC. Specifically, miR-193a-3p affects five key signaling pathways, including DNA damage, Notch, NF-κB, Myc/Max, and oxidative stress, by targeting downstream genes such as SRSF2, PLAU, and HIC2, thereby further enhancing resistance to multiple chemotherapies in BC cells (127). In conclusion, abnormal methylation of related miRNA promoters is closely associated with the proliferation, invasion, migration, and chemotherapy resistance of BC (**Table 2**). By regulating the methylation status of these miRNA promoters, it may be possible to inhibit the development of BC and its associated chemotherapy resistance.

### 5.2 Interaction between miRNAs and DNMTs in BC

**Figure 3:**
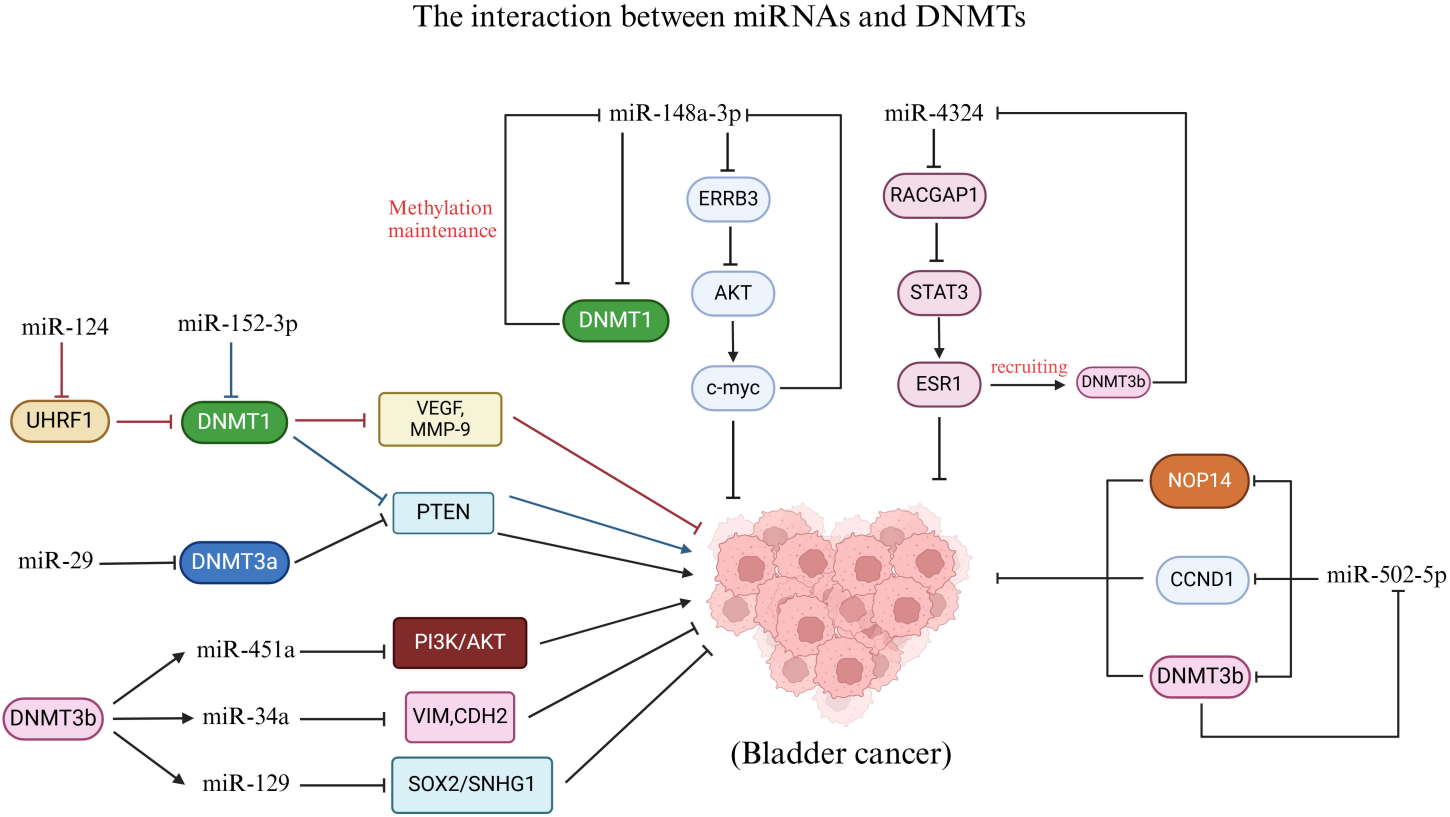
Interaction between miRNAs and DNMTs. MiR-124, miR-152-3p and miR-29 regulate DNMTs to affect BC. DNMT3b affects BC by regulating the methylation status of miR-451a, miR-34a and miR-129. In addition, there is a feedback pathway between miR-148a-3p, miR-4324, and miR-502-5p and DNMTs in the development of BC.

The E3 ubiquitin ligase RING finger domain 1 (UHRF1) serves as a crucial regulator of epigenetic modifications. In the context of DNA methylation, the acetylation of the acetyltransferase Tip60 leads to the degradation of DNMT1. This process triggers the ubiquitination of UHRF1, resulting in the degradation of the DNMT1 protein, which in turn causes abnormal methylation of tumor suppressor gene promoters and promotes cancer development (137, 138). Notably, the expression of miR-124 was found to be inversely correlated with UHRF1 expression in BC, and miR-124 levels were significantly reduced (139). Utilizing bioinformatics prediction algorithms such as TargetScan and miRanda, it was hypothesized that UHRF1 is a potential target of miR-124, a hypothesis that was subsequently validated through a luciferase reporter gene assay. The overexpression of miR-124, which functions as a tumor suppressor in BC, markedly inhibits the expression of angiogenesis markers including VEGF, MMP-2, and MMP-9, thereby suppressing the migration, invasion, and angiogenesis of BC cells *in vitro*, as well as tumor growth *in vivo*. However, restoration of UHRF1 expression can counteract the antitumor effects of miR-124 (139). These findings suggest that miR-124 can inhibit the progression of BC by targeting the DNA methylation factor UHRF1, thereby suppressing the expression of VEGF, MMP-2, and MMP-9. Similarly, miR-29 has been shown to target DNMT3a, influencing the role of the tumor suppressor PTEN in BC (140). In BC, the high expression of ATDCs is closely associated with tumor formation, invasion, and metastasis. Research has demonstrated that ATDCs regulate DNMT3a expression by suppressing the expression of miR-29 family members 29a and 29b, which mediates PTEN silencing and promotes BC proliferation and invasion (140). Additionally, miR-152-3p in BC can also modulate the status and expression of DNA methylation in the PTEN promoter by suppressing DNMT1 expression (141). These findings indicate that miRNAs can regulate the methylation and expression of tumor suppressors by targeting DNA methylation factors (such as UHRF1, DNMT1, and DNMT3a), thereby influencing the progression of BC (**Figure 3**).

Conversely, DNMTs can also regulate miRNA promoter methylation and expression to impact BC. The levels of DNMT3b and miR-34a in BC were demonstrated to be related to the prognosis of patients. Patients with low expression of DNMT3b and high expression of miR-34a had longer overall survival (142). Bisulfite genome sequencing (BSP) analysis revealed that the methylation of the miR-34a promoter in BC tissues with low DNMT3b expression was lower than that in tissues with high DNMT3b expression. Subsequently, *DNMT3b* knockdown cells were generated through lentivirus transfection in BC cell lines. It was observed that the knockdown of *DNMT3b* resulted in reduced methylation of the miR-34a promoter and an increase in miR-34a expression levels. This suggests that DNMT3b may influence the methylation status of the miR-34a promoter. Further investigations indicated that *DNMT3b* knockdown inhibited the downstream targets of miR-34a, specifically hepatocyte nuclear factor 4γ (HNF4γ) and Notch1, leading to decreased migration and invasion of BC cell lines. The reduction in invasive capability was associated with alterations in the expression of EMT-related proteins. Notably, the expression levels of vimentin, N-cadherin, MMP2, and MMP9 decreased, while E-cadherin expression increased (142). In conclusion, DNMT3b silencing inhibits migration and invasion by epigenetically enhancing miR-34a in BC. Isorhapontigenin (ISO) is a novel compound with anticancer properties, and its anticancer mechanism in BC is closely related to that of DNMT3b. Meng et al. reported that ISO treatment significantly inhibited the growth and invasion of MIBC cells in a dose-and time-dependent manner (143). Mechanistic studies have shown that ISO treatment leads to a decrease in the DNMT3b protein level, which mediates the hypomethylation of the miR-129 promoter and enhances its expression. Subsequently, miR-129 regulates SNHG1 to impede tumor progression by transcriptionally inhibiting SOX2. Consequently, ISO can restrain growth and invasion by modulating SNHG1 through the DNMT3b/miR-129/SOX2 axis (143). Additionally, the overexpression of DNMT3b in BC facilitated the methylation of the miR-451a promoter, which resulted in the inhibition of miR-451a and then activated the PI3K/AKT signaling axis to promote the growth and metastasis of BC cells (144). These findings indicate that DNMT3b can influence the progression of BC by regulating related miRNAs (**Figure 3**).

On the other hand, the study revealed a complex feedback pathway between miRNAs and DNMTs in BC. Notably, the expression of miR-148a-3p was significantly decreased in BC. However, treatment with 5-aza-dC restored the expression of miR-148a-3p in the BC cell line, suggesting that DNA methylation may play a role in the silencing of miR-148a-3p in BC (145). Following the overexpression of miR-148a-3p in the BC cell line, the levels of DNMT1 mRNA and protein were found to be reduced. The targets of the miRNA were predicted using the TargetScan database, and dual-luciferase assays confirmed that DNMT1 is a direct target of miR-148a-3p. DNMT1 preferentially binds to semimethylated DNA and is capable of maintaining DNA methylation, thereby promoting the methylation of the miR-148a-3p promoter. Consequently, a positive feedback loop may exist between DNMT1 and miR-148a-3p in BC, where DNMT1 induces hypermethylation of the miR-148a-3p promoter, resulting in decreased expression of miR-148a-3p. This reduction alleviates the inhibition of DNMT1’s downstream target, further suppressing miR-148a-3p expression (145). Interestingly, miR-148a-3p also inhibits BC cell proliferation, migration, and EMT by targeting ERBB3 and AKT2. The ERBB3 transmembrane receptor, a member of the human epidermal growth factor receptor (EGFR) family, plays a role in cell proliferation, migration, and survival upon activation (145). Mechanistically, after miR-148a-3p targets ERBB3, the expression of ERBB3 decreases, leading to reduced levels of phosphorylated AKT, N-cadherin, and fibronectin. AKT phosphorylation activates c-myc, indicating that miR-148a-3p inhibits BC progression through the ERBB3/AKT2/c-myc pathway. Notably, there is a c-myc binding site located in the 600 bp upstream region of the miR-148a-3p transcription start site (TSS) (TGGTCACGTGGC). Consequently, c-myc overexpression suppresses miR-148a-3p, indicating the presence of a feedback loop between c-myc and miR-148a-3p. In conclusion, this study reveals a feedback loop involving miR-148a-3p, DNMT1, and the ERBB3/AKT2/c-myc pathways in BC. Targeting miR-148a-3p may provide potential therapeutic targets for effective BC treatment (145). Similarly, hypermethylation of CpG islands in BC leads to the downregulation of miR-502-5p. The overexpression of miR-502-5p inhibits cell proliferation and migration *in vitro*, as well as tumor growth *in vivo* (21). CCND1, DNMT3b, and NOP14 have been identified as direct targets of miR-502-5p. When miR-502-5p is downregulated, the expression of its downstream target DNMT3b increases, which in turn enhances the methylation of the miR-502-5p CpG island and further downregulates miR-502-5p. Consequently, DNMT3b and miR-502-5p establish a positive feedback loop in the regulation of BC (21). Additionally, the miR-4324/RACGAP1/STAT3/ESR1 feedback loop may serve as a key regulator in the progression of BC (146). The overexpression of miR-4324 retards the proliferation and metastasis of cancer cells by inhibiting the oncogenic protein RACGAP1. ESR1 promotes the expression of miR-4324; however, it is often downregulated in BC due to promoter hypermethylation, which impedes the upregulation of miR-4324. The binding of p-STAT3 to the ESR1 promoter induces the recruitment of DNMT3b, resulting in the methylation of the ESR1 promoter and further downregulation of both ESR1 and miR-4324. Consequently, miR-4324 is part of a complex feedback loop regulatory network in BC, which may enhance its anticancer effect by restoring ESR1 expression (146). In conclusion, there is a complex regulatory network between miRNAs and DNMTs during the progression of BC (**Figure 3**). An in-depth study of the mutual regulatory mechanism between the two plays a crucial role in controlling the progression of BC and developing new therapeutic targets in the future.

## 6 Limitations and future perspectives

**Figure 4:**
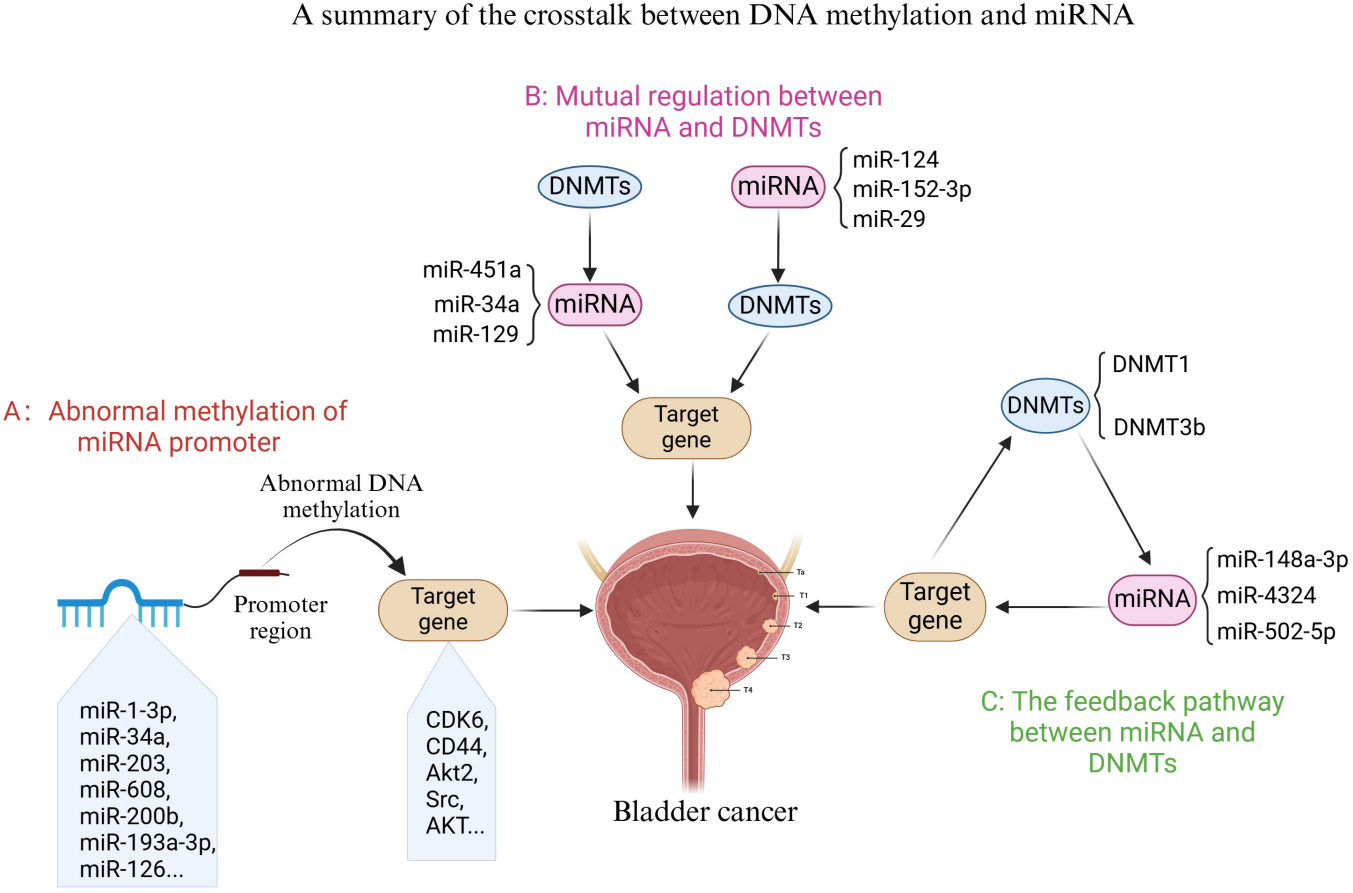
A summary of the crosstalk between DNA methylation and miRNA. As illustrated in panel **(A)** of the figure, certain miRNAs exhibit altered expression as a result of abnormal DNA methylation in the promoter region, which subsequently regulates downstream target genes and influences BC progression. panel **(B)** highlights the reciprocal regulation between miRNAs and DNMTs (DNMTs) during BC progression. Finally, panel **(C)** indicates the presence of a feedback loop involving specific miRNAs and DNMTs in the regulation of target genes associated with BC.

Previous reviews have primarily centered on DNA methylation and/or miRNA as biomarkers for the detection and diagnosis of BC (147, 148). However, there is a notable absence of discussion regarding the complex crosstalk mechanisms between these two factors and their impact on the progression of BC. To the best of our knowledge, this review provides a comprehensive summary of the intricate interactions between DNA methylation status and miRNA expression in BC. As illustrated in **Figure 4**, there exists a complex interplay between DNA methylation and miRNAs, which plays a crucial role in the malignant transformation processes of BC and influences cancer cell proliferation, the cell cycle, migration, invasion, and drug resistance.

Abnormal DNA methylation of miRNA promoters significantly influences BC. As illustrated in **Table 2**, hypermethylation of most miRNA promoters leads to their reduced expression. This reduction subsequently promotes the transcription of downstream transcription factors or the transmission of related signaling pathways, thereby affecting BC proliferation, invasion, migration, and drug resistance. Notably, a few miRNAs (miR-193a-3p, miR-888, miR-23a, miR-27a, and miR-145) exhibit hypomethylated promoters. Furthermore, determining the methylation status of relevant miRNA promoters can serve as a biomarker for the diagnosis and prognosis of BC (135, 136). These findings underscore the importance of a comprehensive understanding of the role of abnormal DNA methylation of miRNA promoters in BC for both preclinical and clinical research. Additionally, there is reciprocal regulation between miRNAs and DNMTs (DNMT1, DNMT3a, and DNMT3b) in BC, encompassing both direct and indirect regulatory mechanisms. On one hand, miRNAs can directly modulate the activity of DNMTs, thereby influencing the function of downstream tumor suppressor genes in BC (e.g., the miR-29/DNMT3b/PTEN axis). Conversely, DNMTs can also regulate miRNA expression, affecting the transcriptional state of downstream targets and consequently influencing BC progression (e.g., the DNMT3b/miR-129/SOX2 axis). On the other hand, as depicted in **Figure 2**, numerous feedback pathways exist between miRNAs and DNMTs, facilitating indirect regulation. Research has demonstrated that most of these feedback pathways, such as those involving DNMT3b and miR-502-5p, are positive. Thus, a strong connection exists between the aforementioned miRNAs and DNA methylation. Targeting these miRNAs or DNA methylation-related factors may offer reliable diagnostic and prognostic biomarkers or promising therapeutic approaches in bladder oncology.

Nevertheless, when interpreting this comprehensive review, several inherent limitations must be acknowledged. First, due to the scarcity of clinical research, the diagnostic, therapeutic, and prognostic significance of miRNAs and DNA methylation in BC requires further investigation. Second, the molecular mechanisms underlying the interaction between DNA methylation and miRNAs in BC need to be clarified and explored. In addition to the currently studied genes and associated signaling pathways, it is essential to consider the role of the tumor microenvironment and tumor immunity in the pathogenesis of BC. Finally, the dual functions of DNA methylation and miRNAs in BC warrant attention. Both abnormal DNA methylation and dysregulated miRNA expression can either promote or inhibit the progression of BC. Despite the technical challenges encountered in applying epigenetic modifications for BC diagnosis, treatment, and prognostic evaluation, the prospects remain promising. Future research will further refine detection methods and delve into the specific mechanisms of epigenetic modifications in BC, providing new strategies for precision medicine. As epigenetic drugs are developed and utilized, epigenetic therapy has the potential to become a significant treatment option for BC.

## 7 Conclusion

On the basis of this review, the interaction between DNA methylation and miRNAs may constitute a crucial molecular mechanism in the onset and progression of BC. On the one hand, adjusting the DNA methylation status of miRNA promoters can affect the transcription of downstream oncogenes or tumor suppressor genes, thus affecting BC. On the other hand, controlling the interaction between miRNAs and DNMTs can also affect BC progression. By attaining a deeper comprehension of the interaction mechanisms between DNA methylation and miRNAs and their specific functions in BC, we may discover more effective diagnostic and therapeutic methods for BC, ultimately resulting in improved quality of life and survival rates for patients.
